# Cystic Echinococcoses in Mongolia: Molecular Identification, Serology and Risk Factors

**DOI:** 10.1371/journal.pntd.0002937

**Published:** 2014-06-19

**Authors:** Akira Ito, Temuulen Dorjsuren, Anu Davaasuren, Tetsuya Yanagida, Yasuhito Sako, Kazuhiro Nakaya, Minoru Nakao, Oyun-Erdene Bat-Ochir, Tsendjav Ayushkhuu, Narantuya Bazarragchaa, Nyamkhuu Gonchigsengee, Tiaoying Li, Gurbadam Agvaandaram, Abmed Davaajav, Chinchuluun Boldbaatar, Gantigmaa Chuluunbaatar

**Affiliations:** 1 Department of Parasitology, Asahikawa Medical University, Asahikawa, Japan; 2 Department of Medical Biology and Histology, School of Biomedicine, Health Sciences University of Mongolia, Ulaanbaatar, Mongolia; 3 National Center for Communicable Diseases, Ulaanbaatar, Mongolia; 4 National Center of Pathology, Ulaanbaatar, Mongolia; 5 National Center for Maternal and Child Health, Ulaanbaatar, Mongolia; 6 Department of Surgery, State Central First Hospital, Ulaanbaatar, Mongolia; 7 Institute of Parasitic Diseases, Sichuan Centers for Disease Control and Prevention, Chengdu, China; 8 Institute of Veterinary Medicine, Ulaanbaatar, Mongolia; 9 Mongolian Academy of Science, Ulaanbaatar, Mongolia; University of Queensland, Australia

## Abstract

**Background:**

Cystic echinococcosis (CE) is a globally distributed cestode zoonosis that causes hepatic cysts. Although *Echinococcus granulosus* sensu stricto (s.s.) is the major causative agent of CE worldwide, recent molecular epidemiological studies have revealed that *E. canadensis* is common in countries where camels are present. One such country is Mongolia.

**Methodology/Principal Findings:**

Forty-three human hepatic CE cases that were confirmed histopathologically at the National Center of Pathology (NCP) in Ulaanbaatar (UB) were identified by analysis of mitochondrial *cox 1* gene as being caused by either *E. canadensis* (n = 31, 72.1%) or *E. granulosus* s.s. (n = 12, 27.9%). The majority of the *E. canadensis* cases were strain G6/7 (29/31, 93.5%). Twenty three haplotypes were identified. Sixteen of 39 CE cases with data on age, sex and province of residence were citizens of UB (41.0%), with 13 of the 16 cases from UB caused by *E. canadensis* (G6/7) (81.3%). Among these 13 cases, nine were children (69.2%). All pediatric cases (n  =  18) were due to *E. canadensis* with 17 of the 18 cases (94.4%) due to strain G6/7. Serum samples were available for 31 of the 43 CE cases, with 22 (71.0%) samples positive by ELISA to recombinant Antigen B8/1 (rAgB). Nine of 10 CE cases caused by *E. granulosus* s.s. (90.0%) and 13 of 20 CE cases by *E. canadensis* (G6/7) (65.0%) were seropositive. The one CE case caused by *E. canadensis* (G10) was seronegative. CE cases caused by *E. granulosus* s.s. showed higher absorbance values (median value 1.131) than those caused by *E. canadensis* (G6/7) (median value 0.106) (*p*  =  0.0137).

**Conclusion/Significance:**

The main species/strains in the study population were *E. canadenis* and *E. granulossus* s.s. with *E. canadensis* the predominant species identified in children. The reason why *E. canadensis* appears to be so common in children is unknown.

## Author Summary

Cystic echinococcosis (CE) is a parasitic zoonosis with a cosmopolitan distribution. Molecular analysis was carried out on 43 hepatic CE cysts from 43 cases confirmed histopathologically at the NCP, Mongolia. Molecular analysis revealed two species, *Echinococcus canadensis* and *Echinococcus granulosus* s.s. Twenty three haplotypes of the *cox1* gene were identified. All pediatric cases (n = 18) were by *E. canadensis*. Sixteen of 39 CE cases with data on age, sex and province of residence were from UB (41.0%), and 13 of these 16 cases were caused by *E. canadensis* (81.3%). Among the 13 cases from UB, nine were children (69.2%). A total of 31 serum samples from these 43 cases were analyzed for antibody response to rAgB with 22 (71.0%) samples positive by ELISA to rAgB. Thirteen of 20 *E. canadensis* (G6/7) (65%) and nine of 10 *E. granulosus* s.s. (90%) were seropositive. CE cases by *E. granulosus* s.s. showed a higher absorbance value than cases by *E. canadensis* (*p*  =  0.0137). This is the first study to evaluate age distribution of and antibody responses to rAgB in CE cases caused by the two species in Mongolia. It remains unknown why *E. canadensis* appears to be more common in pediatric cases.

## Introduction

Cystic echinococcosis (CE) is a globally distributed parasitic zoonosis caused by ingestion of the eggs of *Echinococcus granulosus* sensu lato (s.l.) [1]–[5]. Recent molecular re-evaluation of *E. granulosus* s.l. has revealed that it consists of 5 independent species, *E. granulosus* sensu stricto (s.s.) (G1–G3), *E. equinus* (G4), *E. ortleppi* (G5), *E. canadensis* (G6–G10) and *E. felidis* [4]–[6]. Although *E. granulosus* s.s. (G1) is the major causative agent of human CE where sheep are grazed with dogs [6]–[11], recent molecular studies of human CE specimens have revealed that CE cases caused by *E. canadensis* (G6–G10) are common in some areas where camels and other livestock including cattle, pigs, and goats are distributed [4]–[6], [10]–[22]. Therefore, it is important to include molecular identification of human CE cases in epidemiological studies.

In Mongolia, more than 50% of the population lives in the capital city of Ulaanbaatar (UB), with the remainder largely following the traditional nomadic lifestyle [23]–[28]. For many years, CE has been recognized as a common disease in Mongolia even though there is very little published data. Since the collapse of the Soviet Union in 1991, two meetings have been held in UB on the topic of CE. The first meeting was held at the National Center of Communicable Diseases (NCCD) in May 1995 and Tsoodol, Narantuya and Goosh from Mongolia provided overviews of human cases to date [29]. During this meeting, it was reported that in 1950, 7.8% of all surgical patients were diagnosed CE, whereas this value was only 1.9% in 1990. CE was determined to be the cause of 18% of the surgical cases seen at State Central First Hospital (SCFH) in 1993 [25]–[28]. The second meeting was held at the Health Science University of Mongolia (HSUM) in June 2009 for the purpose of establishing a network of CE experts [27]. During this meeting, Ayushkhuu from the National Center for Maternal and Child Health (NCMCH) summarized 25 pediatric CE cases from 2008 and 2009 (19 cases in 2008 and 6 cases from Jan to May 2009). These cases consisted of 15 boys and 10 girls, including 4 children under the age of 5-years [27]. During this same meeting, Bazarragchaa from the SCFH summarized a total of 144 (63 males and 81 females) CE cases (1989–2009 June) [27]. None of the reported cases differentiate between infection with *E. granulosus* s.s. and *E. canadensis*.

There have been several sero-epidemiology studies of CE in Mongolia [30]–[33]. These studies all have used hydatid cyst fluid (HCF) for screening with an ELISA. However, none of the studies used a confirmatory test to verify their findings. Most surgical cases in Mongolia are diagnosed by histopathological identification of resected lesions at the National Center of Pathology (NCP) in UB [27], [28]. The present study is the first to identify the primary species/strains of CE present in Mongolian patients that had been histopathologically confirmed as having CE as well as evaluate antibody responses to the recombinant Antigen B8/1 (rAgB) by ELISA [34]–[43].

## Methods

### Ethical statement

Molecular analysis of human specimens and serological analysis of antibody responses, and junior researchers from several institutes in UB, Mongolia, to do laboratory analysis of these all specimens were approved by the Asahikawa Medical University (AMU) Institutional Review Board (AMU-IRB-1435).

### Patient samples

A total of 43 CE cases were evaluated, consisting of 18 pediatric cases from the NCMCH and 27 cases from the SCFH. In total, 43 hepatic CE cysts (1 cyst per patient) were obtained and confirmed to be CE histopathologically at the NCP. Pre-surgical serum samples were available for 31 of the 43 study patients (Table 1). De-identified data on patient age, sex, and province of residence were also obtained.

**Table 1:** Molecular identification of the causative species of 43 CE cases and antibody response to rAgB in ELISA (n = 31, cut-off value: 0.055).

Case No.	Province	Age in years (sex)	ELISA (OD value)	PCR	Cox1 haplotype (Accession Nos.)
1	UB#	5 (F)	0.802	*E. canadensis* (G6/7)	EcMGL5 (AB893252)
2	Selenge	21 (F)	x	*E. canadensis* (G6/7)	EcMGL2 (AB893253)
3	UB, Nalaih	4 (F)	0.011	*E. canadensis* (G6/7)	EcMGL2 (AB893253)
4	UB	13 (F)	x	*E. canadensis* (G6/7)	EcMGL6 (AB893254)
5	x	x	0.071	*E. canadensis* (G6/7)	EcMGL7 (AB893255)
6	Umnogovi	8 (M)	0.875	*E. canadensis* (G6/7)	EcMGL2 (AB893253)
7	Selenge	28 (M)	x	*E. granulosus* (G1)	EgMGL1 (AB893242)
8	UB	38 (F)	0.019	*E. canadensis* (G6/7)	EcMGL1* (AB813182)
9	Zavkhan	65 (F)	1.263	*E. granulosus* (G1)	EgMGL2 (AB893243)
10	Arkhangai	50 (M)	x	*E. granulosus* (G1)	EgMGL3 (AB893244)
11	x	15 (M)	x	*E. canadensis* (G10)	EcMGL4 (AB893264)
12	Selenge	62 (F)	0.051	*E. granulosus* (G1)	EgMGL4 (AB893245)
13	Dundgovi	12 (F)	0.661	*E. canadensis* (G6/7)	EcMGL8 (AB893256)
14	Tuv	56 (M)	0.023	*E. canadensis* (G10)	EcMGL4 (AB893264)
15	Dornogovi	22 (F)	0.142	*E. canadensis* (G6/7)	EcMGL9 (AB893257)
16	UB	72 (M)	1.267	*E. granulosus* (G1)	EgMGL5 (AB893246)
17	Bayan-Ulgii	48 (M)	0.273	*E. granulosus* (G1)	EgMGL6 (AB893247)
18	Selenge	68 (F)	1.472	*E. granulosus* (G1)	EgMGL7 (AB893248)
19	Umnugovi	41 (F)	0.003	*E. canadensis* (G6/7)	EcMGL2 (AB893253)
20	UB	4 (M)	0.771	*E. canadensis* (G6/7)	EcMGL1* (AB813182)
21	UB	9 (M)	0.081	*E. canadensis* (G6/7)	EcMGL15 (AB893263)
22	UB	25 (M)	0.261	*E. granulosus* (G1)	EgMGL8 (AB893249)
23	Khuvsgul	10 (F)	0.025	*E. canadensis* (G6/7)	EcMGL10 (AB893258)
24	UB	58 (F)	0.101	*E. canadensis* (G6/7)	EcMGL10 (AB893258)
25	UB	4 (M)	x	*E. canadensis* (G6/7)	EcMGL2 (AB893253)
26	x	13 (M)	1.386	*E. canadensis* (G6/7)	EcMGL11 (AB893259)
27	UB	5 (F)	0.011	*E. canadensis* (G6/7)	EcMGL2 (AB893253)
28	UB	43 (F)	x	*E. canadensis* (G6/7)	EcMGL7 (AB893255)
29	UB	35 (F)	1.024	*E. granulosus* (G1)	EgMGL10 (AB893251)
30	Khovd	46 (F)	x	*E. canadensis* (G6/7)	EcMGL1* (AB813182)
31	Dornogovi	5 (M)	1.301	*E. canadensis* (G6/7)	EcMGL2 (AB893253)
32	UB	15 (M)	x	*E. canadensis* (G6/7)	EcMGL6 (AB893254)
33	Dundgovi	16 (M)	0.036	*E. canadensis* (G6/7)	EcMGL2 (AB893253)
34	Uvurkhangai	59 (M)	0.821	*E. granulosus* (G1)	EgMGL6 (AB893247)
35	Govi-Altai	54 (M)	x	*E. canadensis* (G6/7)	EcMGL2 (AB893253)
36	Selenge	14 (M)	1.396	*E. canadensis* (G6/7)	EcMGL12 (AB893260)
37	Khuvsgul	38 (F)	1.387	*E. granulosus* (G1)	EgMGL9 (AB893250)
38	x	x	x	*E. canadensis* (G6/7)	EcMGL2 (AB893253)
39	Darkhan	29 (M)	0.007	*E. canadensis* (G6/7)	EcMGL2 (AB893253)
40	UB	79 (F)	0.145	*E. canadensis* (G6/7)	EcMGL13 (AB893261)
41	Tuv	21 (F)	1.238	*E. granulosus* (G1)	EgMGL6 (AB893247)
42	UB	4 (M)	x	*E. canadensis* (G6/7)	EcMGL6 (AB893254)
43	Uvurkhangai	9 (F)	0.111	*E. canadensis* (G6/7)	EcMGL14 (AB893262)

x, not available;

*Only partial sequence (828 bp) was obtained.

# UB: Ulaanbaatar (Capital city)

### DNA analysis

Genomic DNA was extracted from 43 ethanol-fixed samples from hepatic CE patients (Table 1) using a DNeasy tissue kit (Qiagen, Hilden, Germany) according to the manufacturer's instructions. The extracted DNA was kept at −20°C until further analysis could be performed. DNA obtained was used as templates for polymerase chain reaction (PCR). The complete or partial mitochondrial cytochrome c oxidase subunit I (*cox 1*) gene was amplified by PCR as reported previously [44]–[46]. PCR products were treated with illustra ExoStar (GE Healthcare) to remove excess primers and dNTPs, and directly sequenced with a BigDye Terminator v3.1 and a 3500 DNA sequencer (Life Technologies). Obtained sequences were edited using Geneious Pro version 7.0.4 (created by Biomatters, available from http://www.geneious.com), and multiple alignments of each *cox1* haplotype were made by the program MAFFT [47] with the homologous sequences of other *Echinococcus* species available in the GenBank database. A phylogenetic tree was constructed using the neighbour-joining method and Kimura's two-parameter model [48] in Phylogenetic Analysis Using Parsimony (PAUP) version 4.0b [49]. The robustness of the phylogenetic tree was tested by bootstrapping with 1000 replicates. For tree construction, *Versteria mustelae* was used as an outgroup because it is a sister to all members of the genus *Echinococcus* [50].

### Serology

Recombinant Antigen B8/1 (rAgB) produced from *E. multilocularis* (rEmAgB) was applied for ELISA and immunoblot tests as reported previously [36]. Several specimens with optical density (OD) values around the cut-off were re-checked by immunoblot using the same antigen for confirmation (figure not shown) [36], [40], [41]. ELISA was carried out in flat-bottom 96-well microplates (Nunc, Maxisorp, Roskilde, Denmark) as previously described [36]. The microplates were coated with 100 ng/ml of rEmAgB diluted in phosphate-buffered saline (PBS) and incubated at 4 °C overnight. Excess antigen was removed by washing with PBS. Blocking was performed with blocking solution [1% casein in 20 mM Tris-HCl (pH 7.6) containing 150 mM NaCl] and the plates were incubated at 37 °C for 1 hr. The plates were washed twice with PBS containing 0.05% Tween 20 (PBST). 100 µl of diluted sera (1/100 dilution in blocking solution) was added and plates were incubated at 37 °C for 1.5 hrs. After washing five times with PBST, 100 µl of protein G-peroxidase conjugate (1/4000 dilution in blocking solution) (Invitrogen, Camarillo, CA) was added to each well and the plates were incubated at 37 °C for 1.5 hrs. Plates were washed six times with PBST and one time with PBS and incubated with 100 µl of substrate solution [0.4 mM 2,2-azino-di (3-ethyl-benzthiazoline-6-sulfonate) (ABTS) in 0.2 M citric acid buffer (pH 4.7)] at room temperature for 30 min. The color reaction was stopped by addition of 1% SDS. The optical density at 405 nm (OD_405_) was determined using an ELISA plate reader (Immuno Mini NJ2300, Tokyo, Japan). The cut-off point was set as the mean OD_405_ plus 3SD for 30 negative control samples.

### Statistical analyses

ELISA OD value results and positive ratios in CE cases caused by *E. granulosus* s.s. (G1) and *E. canadensis* (G6/7) were assessed by the Wilcoxon rank sum test and Fisher's exact test, respectively. All analyses were two-tailed and a *p*-value < 0.05 was considered statistically significant.

## Results

### Molecular identification of *Echinococcus* species causing CE

**Figure 1:**
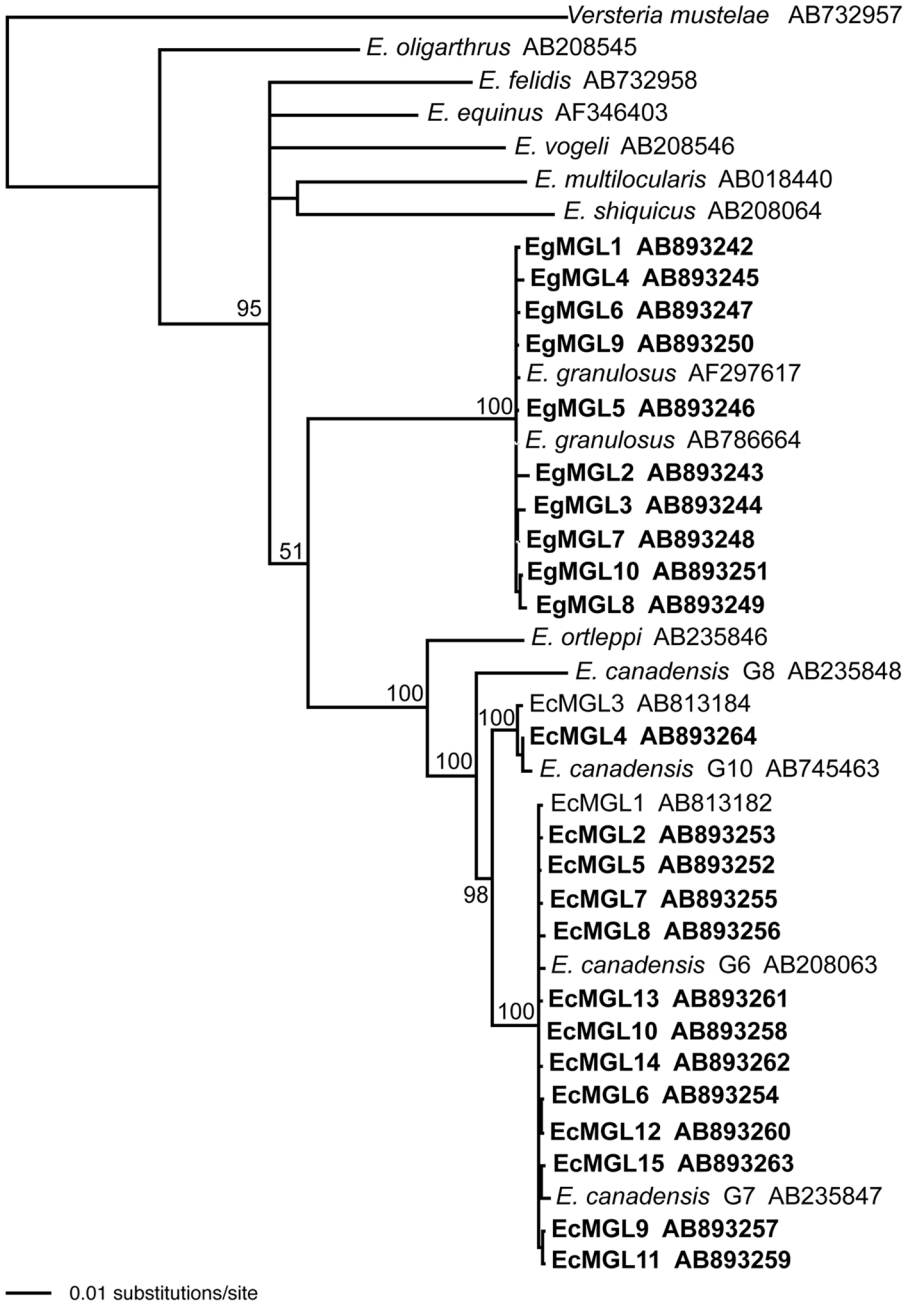
A neighbour-joining tree of *Echinococcus* spp. constructed from the nucleotide sequences of mitochondrial *cox1* gene. Numbers on the nodes are bootstrap values. The names of the haplotypes obtained in this study are shown in bold.

**Figure 2:**
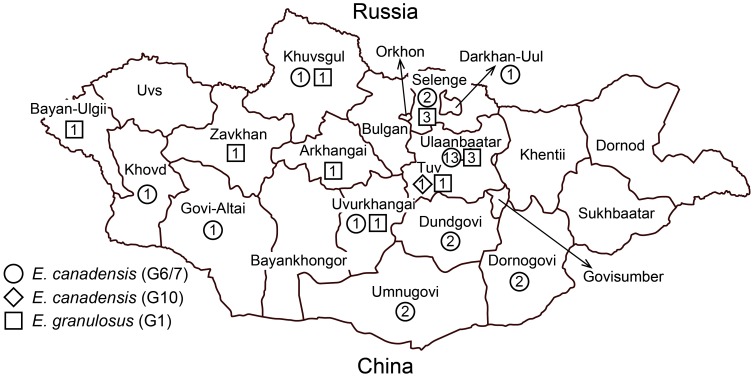
A map of Mongolia showing the distribution of 39 CE cases caused by *E. canadensis* (G6/7) (n = 26), *E. canadensis* (G10) (n = 1), and *E. granulosus* s.s. (G1) (n = 12). Additional four cases had no record of province (see Table 1).

Data on patient age, sex, and province of residence are shown in Table1. Nucleotide sequences of the *cox1* gene (1608-1609 bp) were determined for 40 specimens, and consequently 23 haplotypes were obtained. Among these, 21 haplotypes (EgMGL1-10, EcMGL5-15) were newly identified in Mongolia. The nucleotide sequences of all haplotypes were deposited into DDBJ/EMBL/GenBank databases under the accession numbers AB893242-AB893264. Phylogenetic analysis clearly showed that 12 specimens (10 haplotype) were *E. granulosus* s.s. (G1) and 29 (12 haplotypes) and 2 (1 haplotype) specimens were *E. canadensis* (G6/7) and (G10), respectively (Table 1, Figure 1). For three specimens (Nos. 19, 27, 45), only a partial sequence (828 bp) was determined. BLAST search revealed that the sequence was 100% identical to the *cox1* gene sequence of *E. canadensis* (G6/7) (Accession Number  =  AB813182). Among 43 specimens, 39 [11 *E. granulosus* s.s. (G1), 27 *E. canadensis* (G6/7), one *E. canadensis* (G10)] had data available on patient age, sex and province of residence (Table 1, Figure 2). CE cases were found from 14 provinces, including UB (Figure 2). As shown in Table 1, all pediatric CE cases (n = 18) were caused by *E. canadensis* (100%). Of the 16 CE cases from UB (16/39, 41.0%), 13 cases were caused by *E. canadensis* (G6/7) (13/16, 81.3%). Among these 13 cases caused by *E. canadensis* (G6/7), nine were children (9/13, 69.2%).

### Antibody responses in CE cases caused by *E. granulosus* and *E. canadensis*

**Figure 3:**
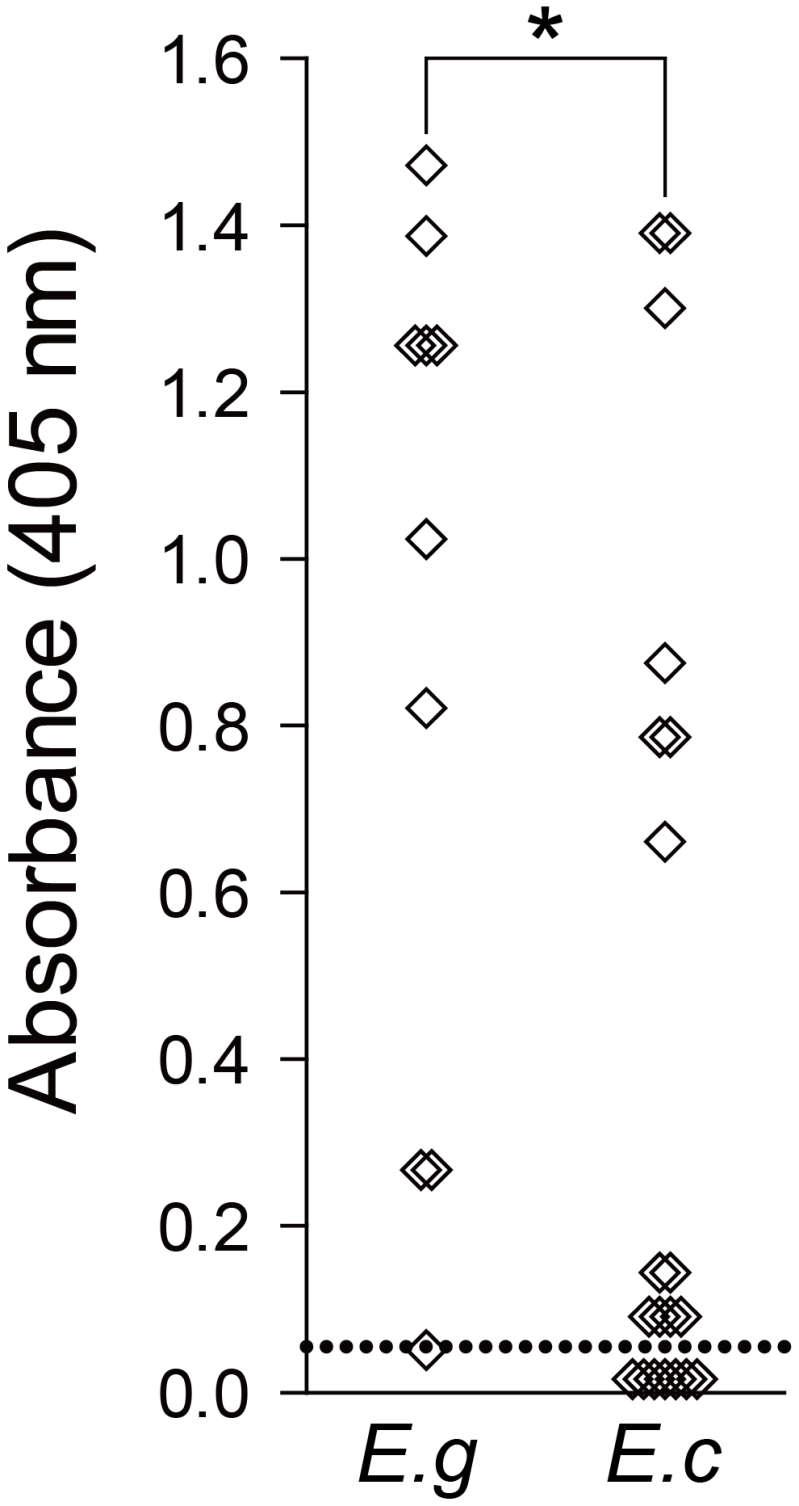
Results of ELISA with rEmAgB for CE cases infected with *E. granulosus* s.s. (G1) (n  =  10) and *E. canadensis* (G6/7) (n  =  20). The cut-off OD value was 0.055, and was indicated by the dotted line. *E.g*:: *E. granulosus* s.s. (G1) case serum samples, *E.c*.: *E. canadensis* (G6/7) case serum samples. *: *p*  =  0.0137

Among 31 serum samples examined, 22 samples (64.7%) were antibody positive to rEmAgB: 9 of 10 *E. granulosus* s.s. (G1) (90%) cases and 13 of 20 *E. canadensis* (G6/7) (65%) cases. The one *E. canadensis* (G10) case was sero-negative (Table 1). Figure 3 illustrates antibody responses of these 30 serum samples to rEmAgB by ELISA. The median absorbance ratios were 1.131 for *E. granulosus* (G1) and 0.106 for *E. canadensis* (G6/7). The *p*-values for sero-positive ratio and absorbance value between the two species were 0.2103 and 0.0137, respectively. The average ages of all CE patients were 47.6 years (range: 21 to 72 years) and 22.5 years (range: 4 to 79 years) for patients infected with *E. granulosus* s.s. (G1) and *E. canadensis*, respectively.

Of the 9 CE cases caused by *E. granulosus* s.s. (G1) with positive serology, 5 were females. Of the 12 CE cases caused by *E. canadensis* (G6/7) with positive serology, 6 were females. CE cases with the highest absorbance values (>1.200) for *E. canadensis* (G6/7) were male children, whereas cases infected with *E. granulosus* s.s. (G1) were all adults (Table 1).

## Discussion

This is the first report on antibody responses to rAgB in patients with CE caused by *E. canadensis*. As shown in Figure 3, nine of 10 *E. granulosus* s.s. (G1) (90%) and 13 of 20 *E. canadensis* (G6/7) (65%) were antibody positive to rEmAgB. While there was no statistically significant difference in the antibody positive ratio between the two species, CE cases caused by *E. granulosus* s.s. (G1) had higher absorbance values (median ratio  =  1.131) than CE cases caused by *E. canadensis* (G6/7) (median ratio  =  0.106) (*p*  =  0.0137). Therefore, the question remains if there is a difference in antigenicity of rAgBs produced by different species of the genus *Echinococcus* or in the antibody response to rAgB in CE patients infected with different *Echinococcus* species. The potential influences of other factors such as pathological stages of CE or differences in health conditions not examined in the present study remain unknown [1]–[3].

The Antigen B8/1 gene has high homology in amino acid sequences (92.6%) for *E. granulosus* s.s. (G1) (EgAgB) and *E. multilocularis* (EmAgB) and has been shown to have the same sensitivity and specificity for detecting CE cases caused by *E. granulosus* s.s. (G1) [34]–[43], . For example, a study in Italy [53] showed that there was no difference in sensitivity or specificity using rEgAgB versus rEmAgB to detect CE cases [36]. The merit to use rEmAgB over rEgAgB is much higher yield performance (Sako et al. unpublished). More critical comparative biochemical and molecular studies [54]–[61] have shown that the amino acid sequence of the epitope region of AgB is well preserved between *E. granulosus* s.s. (G1) and *E. canadensis* (G6/7), with minor difference between *E. granulosus* s.s. (G1) and *E. multilocularis*. Minor differences in amino acid sequences at the N-terminal do not result in any conformational change in the rAgB epitope itself [36], leading to the belief that rAgB prepared from any *Echinococcus* species is useful for detection of antibody response in CE. Serological studies from Australia, France, China, Jordan, Turkey, the United States, Nepal, Poland, Italy, and Iran using rEmAgB have all successfully identified cases of CE [34], [36], [41], [53]. In retrospect, CE cases provided from different countries should have been evaluated for both *E. granulosus* s.s. and *E. canadensis*. Additional comparative studies using both rEgAgB and rEcAgB (*E. canadensis*), rEc6/7AgB or rEc10AgB, to detect CE caused by *E. canadensis* may help confirmation that no difference in sensitivity and specificity exists between rAgBs from different *Echinococcus* species [56].

If we assume that the differences in absorbance values between *E. granlosus* s.s. and *E. canadensis* were not due to a difference in the epitope of rAgBs, one possible cause is a difference in the expression rate of AgB in CE metacestodes [58], which then influences the production of antibodies. As summarized previously [1]–[3], [34]–[43], [51]–[53], antibody response to rAgB differs by the stage of the larval parasite. The response is poor for the cystic lesion (CL) stage as well as inactive cysts (CE4–5), but higher in active and transitional cysts (CE1–3) [51], [53]. Sufficient data were not available from the current study to identify cyst stage for the included patients. However, the present study suggests that abdominal ultrasound alone may not be sufficient to differentiate the *Echinococcus* species responsible for CE [1]–[3] and additional blinded studies are needed to determine if differences can be detected on ultrasound examination [62].

The two species of *E. granulosus* s.l. found in the present study have already been reported from Mongolia by Jabbar et al. [17], however, with opposite results on the predominant species. Jabbar et al. [17] found that 34 of 50 CE cases (68%) were due to infection with *E. granulosus* s.s. (G1–G3) and identified one haplotype of *E. granulosus* s.s. and three haplotypes of *E. canadensis* (G6–G10). The CE cases from the Jabbar et al. [17] were primarily from the eastern part of Mongolia, with no cases from UB. In contrast, CE cases evaluated in the present study were from the central and western parts of Mongolia (Figure 2). Among 39 CE cases with age, sex and province of residence, in the current study, 16 cases (41.0%) were from UB. As more than 50% of the population of Mongolia lives in UB, this was not unexpected. Recent studies have revealed that wolves act as a definitive host of *E. canadensis* (G6/7 and G10) in Mongolia [63]. Wolves are common in the mountainous areas around UB [63]. These mountainous areas are popular holiday destinations for the population of UB. It is, therefore, possible that these individuals are at higher risk for contracting the condition.

The population of Mongolia has close interactions with dogs due, in part, to their nomadic lifestyle. Each household typically has at least one dog, which is usually not tied and is often left to hunt for its own food during the summer months. In these situations, dogs will hunt small mammals and scavenge offal from domestic livestock. It is also common for herdsmen to train dogs to hunt marmots (*Marmota sibirica*), which could potentially be an intermediate host for *Echinococcus* spp. in this region [63]. There is no data from domestic dogs in Mongolia. Therefore, studies on both stray and domestic dogs are needed to better understanding risk factors for human infection. Antibody responses to rEmAgB have been confirmed in cattle, goats, and sheep in Mongolia [64]. Additional studies on antibody responses in camels are needed as is molecular identification of CE cysts in domesticated animals (for example, sheep, goats, cattle, camels) and wild herbivores and omnivores with the potential to act as intermediate hosts.

It is thoroughly unknown why children were over-represented as cases of *E. canadensis* in the current study. If it is assumed that children have access to the same contamination source as adults, one hypothesis is that CE due to *E. canadensis* may become symptomatic much faster than CE due to *E. granulosus* s.s. Further studies, with incorporate sociology and animal ethology components are needed to better understand the transmission ecology of CE caused by *E. granulosus* s.s. and *E. canadensis* in Mongolia.

### Conclusion

Molecular identification of the causative species is important for epidemiological studies on CE. Use of rAgB serology for preoperative and postoperative diagnoses of CE may be a good complement to diagnostic imaging. Further studies are needed to explore the age variation in CE cases caused by *E. granulosus* s.s. versus *E. canadensis* in Mongolia and all other countries where both species are co-distributed. Additional studies on human CE cases, intermediate hosts, and definitive hosts are needed to better evaluate the epidemiology of the circulating *Echinococcus* species as well as risk factors for infection.

#### Accession numbers of *cox1* gene sequcences of *Echinococcus granulosus* s.s. and *Echinococcus canadensis*

The nucleotide sequences of all haplotypes reported in the present study are available in DDBJ/EMBL/GenBank database under the accession numbers AB893242-AB893264.
